# Feasibility of Reviewing Digital Food Images for Dietary Assessment among Nutrition Professionals

**DOI:** 10.3390/nu10080984

**Published:** 2018-07-27

**Authors:** Ayob Ainaa Fatehah, Bee Koon Poh, Safii Nik Shanita, Jyh Eiin Wong

**Affiliations:** 1 Nutritional Sciences Programme & Center for Community Health, Faculty of Health Sciences, Universiti Kebangsaan Malaysia, Kuala Lumpur 50300, Malaysia; ainaa.ayob@gmail.com (A.A.F.); pbkoon@ukm.edu.my (B.K.P.); 2 Dietetics Programme & Center for Community Health, Faculty of Health Sciences, Universiti Kebangsaan Malaysia, Kuala Lumpur 50300, Malaysia; nikshanita@ukm.edu.my

**Keywords:** digital food image, portion size estimation, dietary assessment, nutritionist, dietitian

## Abstract

Validity of image-assisted and image-based dietary assessment methods relies on the accuracy of portion size estimation based on food images. However, little is known on the ability of nutrition professionals in assessing dietary intake based on digital food images. This study aims to examine the ability of nutrition professionals in reviewing food images with regard to food item identification and portion size estimation. Thirty-eight nutritionists, dietitians, and nutrition researchers participated in this study. Through an online questionnaire, participants’ accuracy in identifying food items and estimating portion sizes of two sets of digital food images presenting a meal on a plate (Image PL) and in a bowl (Image BW) were tested. Participants reported higher accuracy in interpreting Image BW compared to Image PL, both in terms of accuracy in food identification (75.3 ± 17.6 vs. 68.9 ± 17.1%) and percentage difference in portion size estimation (44.3 ± 16.6 vs. 47.6 ± 21.2%). Weight of raw vegetables was significantly underestimated (−45.1 ± 22.8% vs. −21.2 ± 37.4%), while drink was significantly overestimated (40.1 ± 45.8% vs. 26.1 ± 32.2) in both images. Less than one-third of the participants estimated portion size within 10% of actual weight for Image PL (23.7%) and Image BW (32.3%). Accuracy of nutrition professionals in reviewing food images could be further improved with training on better perception of portion sizes from images.

## 1. Introduction

Food images, including those taken with handheld devices and wearable cameras, have been widely used in dietary assessment methods [1]. Food images are often used to complement or enhance dietary recalls [2] and food records [3], and collectively form image-assisted dietary methods. More recently, digital food images, especially those captured passively by wearable cameras, have been used as the primary tool for dietary records [4,5,6]. These image-based dietary assessment methods have been employed to improve diet recording in free-living settings, and have been shown to be preferred by participants who may use this method to record their diet intake for up to 1.5 months [7].

Image-assisted and image-based dietary assessment methods have been shown to be feasible and accepted by users of different age groups and countries [7,8,9], including in Malaysia [10]. One of the key considerations when establishing these methods lie in the accuracy of reviewing food images. This image review process requires trained human analysts or an automated system to ‘translate’ the captured image into food items and its portion size, and ultimately into nutrient values [1]. While there is advanced development of automated systems to auto-recognize food, estimate portion size and nutrient content [4,11,12,13,14], the existing studies have yet to establish the reliability of these automated systems due to the complexity of foods and meals [6].

The increased application of image-assisted and image-based dietary methods has heightened the need for accurate image review by human analysts. Food portion size estimation by nutritionists, who had undergone training in dietary assessment, specifically in portion size estimation were reported to be most accurate and had less variability compared to those without training [9,15,16]. However, very few studies have examined the reliability of nutrition professionals such as nutritionists or dietitians in assessing dietary intake based on food images [12,17,18,19]. To date, only five studies have examined the accuracy of trained human analysts in reviewing food images for dietary assessment, albeit among nutritionists [15], dietitians [12,20], nutrition students [21], dietetic students and interns [22] in a Western context. The receptiveness of nutrition professionals towards image-assisted or image-based methods and their ability to review food images also remain unknown.

Therefore, the aim of this study was to determine the ability of nutrition professionals in reviewing digital food images taken from image-assisted dietary methods. Specifically, this study seeks to determine the accuracy of nutritionists, dietitians and nutrition-trained researchers in identifying and estimating the serving size of food items served on a plate or in a bowl, as captured in digital food images.

## 2. Materials and Methods

This cross-sectional study was conducted among nutrition professionals in Malaysia. Nutritionists, dietitians, and researchers who had formal university-level training in dietetics, nutrition, and/or food science and whose work involved dietary assessment were eligible for this study. A minimum number of 35 participants was needed to achieve 80% power to detect moderate effects (d = 0.47) based on a priori sample calculation using findings from a previous study [23]. To recruit participants, posters and a dedicated website [24] were set up and advertised in social media groups (Facebook, Instagram and Whatsapp) and through professional contacts. Interested participants were first screened for eligibility through an online form built using a cloud-based survey form software Typeform [25]. Later, eligible participants were each given a unique token through emails and invited to complete a study questionnaire online. The data collection period spanned from December 2015 to April 2016. The study protocol was approved by the Research Ethics Committee of Universiti Kebangsaan Malaysia (NN-2015-084) and performed in accordance with the ethical standards laid down in the 1964 Declaration of Helsinki. All participants provided their consent online before participating in the survey.

### 2.1. Study Questionnaire

The study questionnaire was developed by researchers with technical support by the Department of Information Technology and Learning Support, Universiti Kebangsaan Malaysia using the LimeSurvey online survey tool [26]. There were four versions of the study questionnaire, each consisting of three sections. Each participant was randomly assigned to one of the four versions of the study questionnaire which offered different sets of food images in the first two sections. This random assignment of questionnaire was applied to prevent discussion among participants who were likely to be colleagues within the field.

**Figure 1:**
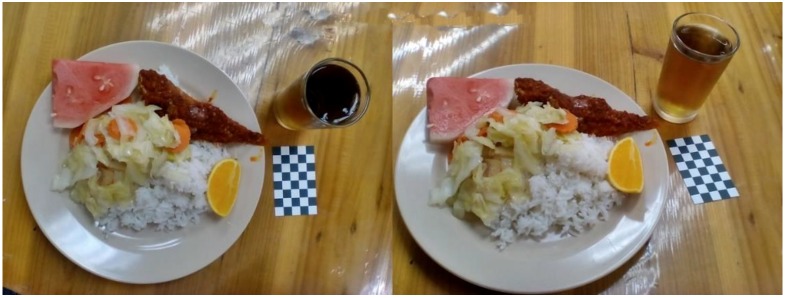
Sample image for Image PL, consisting six items which are the rice, fish, cooked vegetables, watermelon, orange and grass jelly drink. Image on the left was taken at approximately 90° angle and image on the right was taken at approximately 45° angle. Both images were taken in a lunch setting by a participant from a previous study [27].

**Figure 2:**
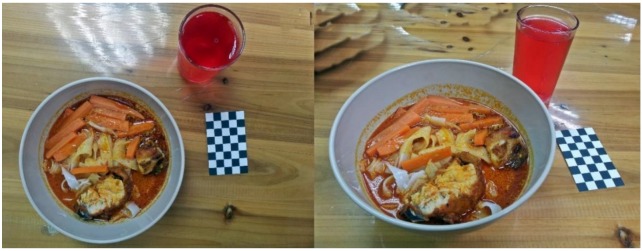
Sample image for Image BW, consisting five items which are rice noodles, curry, chicken, carrots and syrup drink. Image on the left was taken at approximately 90° angle and image on the right was taken at approximately 45° angle. Both images were taken in a lunch setting by a participant from a previous study [27].

In section one, participants were shown the first set of images depicting a meal presented on a plate (Image PL, see Figure 1). In section two, participants were presented with the second set of images which featured foods presented in a bowl (Image BW, see Figure 2). Each set of images comprised two images of a same meal taken from two camera angles: approximately 90° angle and 45° angle which provided aerial and angled views of the food, respectively. In total, participants were presented with 4 images (i.e., 2 sets: Image PL and Image BW) in the first two sections. All images were scaled to dimension of 4000 pixels (width) × 1600 pixels (height) and presented in 72 dpi. Each set of images featured 5 to 7 food items, including a staple (white rice or noodles), protein foods (chicken, fish or egg), vegetables (raw/blanched or cooked), and a drink (plain or flavored). Some images also contained sliced fruits and chili-based sauce (e.g., *sambal*).

All food images were captured by 18- to 20-year-old adult users of an image-assisted food record (in the form of a smartphone application) from a previous study conducted by Chin [27]. In that study, foods were self-served by the participants in a lunch buffet. The weight of each food items were weighed by the researchers using a food weighing scale (Tanita KD-160) and recorded to the closest 1 g. The participants captured the self-served foods using their low to med range Android smartphone cameras before eating. A fiducial marker with black and white checkered design (8 cm × 5 cm) was placed beside the plate or bowl in every image. The fiducial marker was designed following a standard size of a business card as suggested by a previous study [28]. This fiducial marker serves as the size reference and was included to assist in portion size estimation [5,29,30].

In the current study, participants were informed about the actual size of the fiducial marker which appeared in every image. They were asked to list the names of the presented food items, estimate their portion sizes in household measurements and convert to their corresponding weight estimated to the closest 1.0 g or milliliter, as precisely as possible. In the instructions, participants were allowed to use any aids including the fiducial markers provided, food albums, dietary software or food database to assist in their estimation. Participants also were asked questions about challenges during food image interpretation (e.g., ‘In general, did you encounter any difficulties when analyzing this image?’), and their suggestions to overcome the difficulties. Besides, the application of the fiducial marker was also asked through the question ‘Did the checkered card shown in this image help in your estimation?’ together with a description of how it was used.

The last section of the study questionnaire identifies the participants’ receptiveness toward using food images in dietary assessment. Four questions were asked to explore the participants’ current dietary assessment practice at work, and willingness to use digital food images for dietary assessment. Participants were also asked to describe reasons for their willingness or refusal to use this method. Weekly email reminders (once in every 5 to 7 days) were sent to remind participants to complete the online questionnaire within four weeks.

### 2.2. Data Processing and Statistical Analysis

Based on images PL and BW, participants provided responses on the (i) names of the presented food items and drink; and (ii) corresponding portion sizes. The answers provided were compared with the actual food names and weighed portion sizes.

For food identification, participants’ responses were categorized as ‘accurate’, ‘inaccurate’ or ‘omitted’ [31,32]. Accuracy of food identification was determined based on guidelines from previous studies [23,31]. Food items listed by participants that matched with the standard food name (i.e., reference) were considered as ‘accurate’. On the contrary, responses which were either wrong (e.g., fried mackerel instead of fried chicken) or overly generic in description for the food (e.g., mixed vegetables instead of cabbages) were considered ‘inaccurate’. Food items that were not recognized from the food images were recorded as ‘omitted’. The percentage of accuracy for a food item’s identification was calculated by total number of identification over the total actual number of food items.

(1) $$\%\;\mathrm{accurate}\;\mathrm{identification}=100\cdot (\frac{\text{Total accurate number of food items}}{\text{Total actual number of food items}})$$

(2) $$\%\;\mathrm{inaccurate}\;\mathrm{identification}=100\cdot (\frac{\text{Total inaccurate number of food items}}{\text{Total actual number of food items}})$$

(3) $$\%\;\mathrm{omitted}\;\mathrm{food}\;\mathrm{item}=100\cdot (\frac{\text{Total omitted number of food items}}{\text{Total actual number of food items}})$$

To determine the accuracy of portion size estimation, food weight estimated by participants was compared to the actual or ‘ground truth’ weight in matrices unit (1 g or 1 mL). Percentage difference of the estimated and actual weight was calculated using the formula below. Accurate estimation should fall within ±10% difference from actual weight [33]. Estimation with less than 10% difference was considered as underestimated, while more than 10% difference was overestimated.

(4) $$\%\;\mathrm{difference}=100\cdot (\frac{\text{Estimated weight}-\text{Actual weight}}{\text{Actual weight}})$$

All analyses were done using SPSS version 17.0. (SPSS Inc., Chicago, IL, USA). Descriptive statistics were applied to describe demographic characteristics of participants, accuracy of food item identification and portion size estimation, and responses on the image-based dietary assessment method. The mean difference of portion size estimation was calculated using independent *t*-tests, while difference between estimated and actual weight of food item was determined using paired-sample t-test with significance at *p* < 0.05.

## 3. Results

Among 84 nutrition professionals who had registered as participants, only 31 (37%) managed to complete the entire online survey within four weeks. Included in this study was data from 38 participants (16 dietitians, 8 nutritionists and 14 researchers) aged 26.0 ± 0.4 years, including seven participants who had completed only one out of three sections of the study questionnaire. The majority of participants was female and had bachelor degree in Nutritional Sciences, Dietetics, or Food Science and Nutrition, and represented a variety of work settings (Table 1).

### 3.1. Accuracy in Identifying Food Items

More than half of the food items presented in images PL and BW were accurately recognized by the participants. Inaccurate identification of food was found to be more prevalent for foods that were served on a plate (20.9%) than those in a bowl (10.7%) (Table 2). Table 3 illustrates the ability of participants to identify food by food categories. More than half of the estimations were inaccurate for drinks presented in Image PL and noodles with soup and chicken in Image BW. Omission was highest (54.8%) for sauces presented in Image PL.

### 3.2. Accuracy in Estimating Portion Size

Based on the mean difference between the mean estimated and mean actual food weights, raw vegetables were significantly under-estimated (*p* < 0.05), while drinks were significantly over-estimated in both images (*p* < 0.001). The percentage difference ranged from −45.1% (raw vegetables) to 40.1% (drinks) for Image PL; and −21.2% (raw vegetables) to 104.2% (noodles with soup and chicken) for Image BW (Table 4). Mean percentage difference was slightly higher in Image PL (47.6 ± 21.2) compared to Image BW (44.3 ± 16.6). By comparing Image PL and Image BW, mean percentage difference was not significantly different.

Table 5 shows that a higher proportion of participants accurately estimated portion sizes presented in Image BW than those in Image PL (32.3% vs. 23.7%). More than two-thirds of the participants over-estimated portion size presented in Image PL.

### 3.3. Experience in Image-Based Dietary Assessment Method

Table 6 shows the participants’ experience after completing food identification and portion size estimation based on digital food images. The main challenges encountered by participants and the suggestions they provided for improving estimations are summarized in Table 7. All except two of the participants expressed interest in using food images for dietary assessment methods. The reasons provided for disagreeing with the use of image-based dietary assessment method were (i) the concern that taking food images would be too burdensome for the users; and (ii) inconsistent angles when capturing images might affect the perception of food portion size.

## 4. Discussion

The present study examined the ability of young nutrition professionals, comprising practicing nutritionists, dietitians and nutrition-trained researchers from different work settings in Malaysia, in reviewing digital food images for dietary assessment. The findings suggested that although food identification was good, less than one-third of the participants could accurately estimate portion sizes within 10% error based on digital images.

### 4.1. Food Identification

The percentages of food item recognized accurately was found to be higher for foods served in a bowl (Image BW) compared to those served on a plate (Image PL). This difference in food identification accuracy can be explained largely by the repertoire of foods typically served in different serving containers. In Malaysia, a soup-based meal, such as noodles with varying types of soups (example shown in Figure 2, Image BW), are commonly eaten in a bowl. This has narrowed the food item responses for participants, making it easier to make accurate identification from Image BW. Whereas, food items served on a plate are more varied in terms of ingredients and cooking style, and therefore more likely to be misidentified. This issue on the variety of mixed dishes has also been mentioned by Pennington [34], that it is crucial to clarify the ingredients from the food images for an accurate dietary intake analysis. Similarly, the same foods that appeared with different cutting or cooking style in the image might also be confusing even for the experienced personnel [34]. As an example from this study, due to the similar cut and cooking style, participants mistakenly recognized small cuts of ‘fried chicken’ as ‘fried fish’ for Image PL. On the other hand, inaccuracy for image BW was mainly due to errors in distinguishing the types of soups served in the bowl. For instance, participants misidentified ‘tom yum soup’ as ‘curry’ due to the similar color of the spicy soup in the digital images. Besides food, drinks were also commonly misidentified. The inaccuracy of drinks identification was higher in Image PL compared to Image BW. This is due to inconsistency in the types of drinks presented in Image PL while a consistent type of drink was presented in Image BW. A dark-colored drink presented in Image PL, which was ‘grass jelly drink’ and ‘sarsaparilla drink’, was mistakenly recognized as ‘cola’ or ‘iced coffee’. Meanwhile, Image BW presented the same type of drinks, which is the rose syrup drink.

In terms of food omission, drinks (such as drinking water or plain water) and condiments (such as the local spicy sauce, *sambal*) tended to be omitted by participants. Relative to Image PL, more food items presented in a bowl, such as chicken pieces, eggs and raw or blanched vegetables accompanying a soup meal, were omitted in this study. This omission might be caused by the visual presentation of the foods [18,35]. In a bowl, different food items tend to be stacked, hindering some ingredients of a mixed dish or combination food from being clearly seen in the image. These findings corroborate the suggestion from previous studies [34,36,37] to add food labels or textual information to food images to facilitate more accurate food identification, especially for image-assisted food records.

### 4.2. Portion Size Estimation

A higher proportion of participants accurately estimated portion sizes presented in a bowl than those presented on a plate (Table 5). This finding further supports previous observations that a meal portion size estimation is better when estimations are done using a volumetric container rather than dimension [38]. The participants’ responses showed that portion sizes were estimated in volume for liquid-based food items such as beverages, curries, and soups, which were mostly presented in Image BW. Meanwhile, estimation were provided in grams for solid and amorphous food items. Moreover, poorer portion size estimation for plated foods was likely due to the failure to recognize the size of the plate or foods, in terms of dimension. As reported by the participants (Table 6), more than 70% (Image PL) and 65% (Image BW) of participants did not find the fiducial marker provided was helpful in their estimation. It seems possible that participants were unfamiliar with the checkered card provided, and thus were not using the fiducial marker to determine size or dimension from the food images.

White rice and fruits were most accurately estimated among all food items presented in the images. Rice, as the staple for Malaysians, is the common feature in all meals and thus often estimated with accuracy in diet records by the nutrition professionals. The consistent shapes and cuts of foods could also help in accurate portion size estimation of fruits [15,35,39]. Raw vegetables (e.g., cucumber, carrots, bean sprouts) were significantly under-estimated, while drinks and chicken were consistently over-estimated in both images. These findings are consistent with those of Japur and Diez-Garcia [21], which found that there was a trend toward underestimating low-energy foods, and overestimation of high-energy foods among nutrition students.

The participants in this study represent young professionals within the field of nutrition and dietetics and were not given any specific training on analyzing dietary intake based on digital food images. As a result, only 24–33% of the participants could accurately estimate portion size within 10% of actual weight. This quantification accuracy seems to be higher than 18% (of total estimates) reported by Japur and Diez-Garcia [21] among nutrition undergraduates in Brazil, but lower than the 38% reported by Howes and colleagues [22] among dietetics students and interns in the United States and Australia. While one would hypothesize that quantification accuracy is higher among professionals, it is worth noting that only one participant had prior experience in using image-assisted dietary assessment and none in this study had any experience applying fiducial marker in their work. In addition, the use of real-life food images taken in free-living settings may have elevated the level of difficulty in analyzing the images. Individual food items were not organized in any particular manner, which may have affected the participants’ ability to identify and perceive the size of food [40].

### 4.3. Receptiveness Towards Image-Based Dietary Assessment Method

A higher proportion of participants (47.4%, *n* = 18) reported that it was challenging to analyse plated items, compared to the proportion who reported difficulty in analyzing bowled items (25.8%, *n* = 8). This correlated with findings on accuracy, where both food identification and portion size estimation were found to be lower for Image PL. Despite this, a majority of the participants were willing to use digital images in dietary assessment. Feedback from the participants was encouraging, as they reported that using food images in dietary assessment methods may clarify dietary reports by their clients. Food images could also make nutrient analysis more fun and interactive during the consultation process. Furthermore, using food images will reduce the communication barrier between the clients and nutrition professionals. In addition, participants expressed that improving image quality (e.g., a close-up or higher resolution picture) and providing some text information or cues on the images would help in providing better estimation, and hence make the analysis easier. Other than that, a clear description should be provided to teach participants on how to use the provided fiducial marker as a measuring aid when analyzing food images.

To the best of our knowledge, this is the first study to examine the accuracy of identifying food and estimating portion size from digital images among nutrition professionals in an Asian context. The study compared the estimation in bowls and plates, and tested on digital food images taken by adults in another study. This employment of real-life food images is advantageous, as it produces results that simulate the real challenges faced by nutrition professionals when reviewing images in dietary assessment.

Nevertheless, the generalizability of this finding is subject to two main limitations. First, is the low survey completion rate, which resulted in only 38 out of 84 (45%) participants whose data were included in the analysis. The high work commitment and time constraint may have caused a high drop out among the recruited nutrition professionals. This response rate, however, is higher than a previous study on nutritionists [18], and the sample size seems to be larger than the typical sample size (10 to 25 participants) presented in previous nutrition estimation studies conducted among registered nutritionists or dietitians [15,18,20]. We acknowledge that our modest sample size may have limited statistical power to detect small weight differences for some food items. Second, is the young demographic of the sample in terms of age and work experience (maximum 8 years of experience). It is likely that the observed findings may have underestimated the true accuracy of nutrition professionals in Malaysia. Notwithstanding these limitations, the study offers an important and pragmatic evidence on the ability and needs of young nutrition professionals, who will most likely adopt image-based or image-assisted dietary assessment methods in their practice.

## 5. Conclusions

Considering Malaysia as a multi-cultural country with a wide variety of foods and eating practices, identifying food items based on images alone without any information cue may not be an easy task even for the nutrition professionals. Portion size estimations were generally more accurate for food items presented in a bowl rather than those on a plate, with less than one-third of the participants able to estimate portion size within 10% of actual weight. The combination of findings provide support for the need of image-based dietary assessment training for nutrition professionals. Education and hands-on training which focuses on using a suitable fiducial marker and improving perception of portion sizes from food images are recommended.

## Figures and Tables

**Table 1:** Sample characteristics and experience in dietary assessment method.

Sociodemographic	Total (*n* = 38)	Median ± S.E (Min, Max)
	*n* (%)	
Age		26.0 ± 0.4 (24, 36)
Sex		
Male	5 (13.2)	
Female	33 (86.8)	
Highest education level		
Bachelor’s degree	34 (89.5)	
Master’s degree	4 (10.5)	
Current Occupation		
Nutritionist	8 (21.1)	
Dietitian	16 (42.1)	
Researcher	14 (36.8)	
Work setting		
Hospital/health clinic	15 (39.5)	
Sports institute	4 (10.5)	
University	12 (31.6)	
Malaysia Ministry of Health’s headquarters	3 (7.9)	
Research institute	4 (10.5)	
Work duration (months)		12.0 ± 2.9 (3, 96)
≤12 months	25 (65.8)	
>12 months	13 (34.2)	
Experience in portion size estimation (months)		12.0 ± 3.2 (3, 96)
≤12 months	27 (71.1)	
>12 months	11 (28.9)	

**Table 2:** Participants’ accuracy of food item identification for Image PL and Image BW (mean ± sd).

	Image PL			Image BW		
	Number of Food Items Presented	Number of Food Items Identified	Percentage of Food Items Identified ^1^	Number of Food Items Presented	Number of Food Items Identified	Percentage of Food Items Identified
	6.5 ± 0.51			6.5 ± 0.80		
Accurate		4.4 ± 1.15	68.9 ± 17.1		4.9 ± 1.35	75.3 ± 17.6
Inaccurate		1.4 ± 1.11	20.9 ± 15.4		0.7 ± 1.35	10.7 ± 12.4
Omission		0.7 ± 0.70	10.3 ± 11.0		0.9 ± 1.11	14.1 ± 16.8

^1^ The percentage of accuracy for food item identification was calculated for every participant based on the number of food items correctly recognized over the total number of actual food presented. Individual percentage was averaged to produce a mean percentage. Digital food images presenting a meal on a plate (Image PL) and in a bowl (Image BW).

**Table 3:** Accuracy of food item identification based on food categories in Image PL (*n* = 38) and Image BW (*n* = 31).

Food Categories	Total Number of Food Items Presented (*n*)	Accurately Identified (%)	Inaccurately Identified (%)	Omitted (%)
Image PL	208			
Rice	38	38 (100.0)	-	-
Fish	25	22 (88.0)	3 (12.0)	-
Chicken	24	19 (79.2)	5 (20.8)	-
Cooked vegetables	27	16 (59.3)	11 (40.7)	-
Raw vegetables	11	11 (100.0)	-	-
Fruits	14	14 (100.0)	-	-
Sauce	31	11 (35.5)	3 (9.7)	17 (54.8)
Drinks	38	13 (34.2)	20 (52.6)	5 (13.2)
Image BW	139			
Noodles (with soup and chicken)	5	1 (20.0)	4 (80.0)	-
Noodles (with plain soup)	26	16 (61.5)	10 (38.5)	-
Chicken	26	20 (76.9)	4 (15.4)	2 (7.7)
Egg	20	17 (85.0)	1 (5.0)	2 (10.0)
Raw vegetables	31	14 (45.2)	11 (35.5)	6 (19.4)
Drinks	31	28 (90.3)	3 (9.7)	-

**Table 4:** Accuracy of portion size estimation by number of estimation for Image PL (*n* = 38) and Image BW (*n* = 31).

	Total Number of Food Items Presented ^1^ (*n*)	Actual Weight (g)	Number of Estimation ^2^ (*n*)	Estimated Weight (g)	Percentage Difference ^3^ (%)	*p*-Value ^5^
						0.485 ^6^
Image PL	208		190		47.6 ± 21.2 ^4^	
Rice	38	143.3 ± 49.2	38	140.8 ± 70.4	3.5 ± 54.4	0.83
Fish	25	68.4 ± 6.8	25	93.6 ± 32.3	36.5 ± 42.4	<0.001
Chicken	24	49.8 ± 10.2	24	83.1 ± 42.9	64.4 ± 68.5	<0.001
Cooked vegetables	27	71.2 ± 32.5	27	80.9 ± 43.2	16.9 ± 43.5	0.11
Raw vegetables	11	23.0 ± 0.0	11	12.6 ± 5.2	−45.1 ± 22.8	<0.001
Fruits	14	77.5 ± 14.0	14	81.0 ± 52.6	2.6 ± 54.9	0.79
Sauce	31	13.6 ± 8.2	17	18.4 ± 11.4	14.9 ± 49.6	0.59
Drinks	38	175.7 ± 5.5	34	246.2 ± 81.1	40.1 ± 45.8	<0.001
Image BW	139		127		44.3 ± 16.6 ^4^	
Noodles (with soup and chicken)	5	237.0 ± 0.0	5	484.0 ± 118.4	104.2 ± 50.0	0.01
Noodles (with plain soup)	26	298.7 ± 13.10	26	263.0 ± 138.5	−11.0 ± 48.3	0.22
Chicken	26	53.0 ± 19.5	24	67.6 ± 26.7	36.4 ± 65.8	0.05
Egg	20	23.0 ± 0.0	18	26.5 ± 11.6	15.2 ± 50.2	0.22
Raw vegetables	31	68.1 ± 53.4	23	38.8 ± 23.6	−21.2 ± 37.4	0.02
Drinks	31	187.2 ± 9.9	31	235.5 ± 61.5	26.1 ± 32.2	<0.001

^1^ Total number of food items displayed on the food images; ^2^ Total number of food item identified and estimated by the participants; ^3^ Mean percentage difference for each food category = ∑(estimated weight (g) − actual weight (g)/actual weight (g)) × 100 divided by total number of estimation. Accurate estimation should have a percentage difference of ±10%; ^4^ Total mean percentage difference for Image PL and BW were calculated as total absolute percentage difference = (|percentage differences|)/total number of estimations; ^5^ Paired *t*-test was used to calculate the significant difference between actual weight (g) and estimated weight (g) of each food items with a significance at *p* < 0.05. ^6^ Independent *t*-test was used to determine the total absolute mean percent difference between Image PL and Image BW.

**Table 5:** Proportion of participants who accurately estimated portion size ^1^.

	Image PL (*n* = 38)	Image BW (*n* = 31)
	*n* (%)	*n* (%)
Accurate (%)	9 (23.7)	10 (32.3)
Inaccurate (%)	29 (76.3)	21 (67.7)
Under-estimate (%)	3 (7.9)	9 (29.0)
Over-estimate (%)	26 (68.4)	12 (38.7)

^1^ Portion size estimation was considered accurate if percentage difference of the participant is within 10% error.

**Table 6:** Experience in interpreting digital food images.

Variables	*n* (%)
Encounter difficulties when analyzing Image PL (*n* = 38)	18 (47.4)
Encounter difficulties when analyzing Image BW (*n* = 31)	8 (25.8
Checkered card shown in Image PL help in estimation (*n* = 38)	9 (23.7)
Checkered card shown in Image BW help in estimation (*n* = 31)	10 (32.3)
Willing to use digital photography method for dietary assessment (*n* = 31)	29 (93.5)
Current dietary assessment method used (*n* = 31)	
24-h diet recall	10 (32.3)
Food frequency questionnaire	3 (7.9)
Diet history	11 (35.5)
Food record	6 (19.4)
3-day pictorial food record	1 (3.2)

**Table 7:** Challenges encountered and suggestions for improvement given by participants.

Challenges Encountered by Participants	Suggestions for Improvement
Difficult to recognize types of drinks and syrup used.	Provide text description of foods and drinks. Capture image of sugars or syrup separately when used in the drinks preparation.
Foods images are not distinguishable from other food items.	Make sure the different food items were not stacked and can be seen clearly. Use different plates to segregate different food items.
Image quality was unsatisfactory (low resolution)	Reduce the distance between the camera and plate to produce a clearer image.
Difficult to recognize the size of the plate and bowl.	State the depth or volume of the bowl. Provide a ruler in the image to show the diameter of the plate and bowl.
